# Adiponectin and Polycystic Ovary Syndrome in Adolescent Girls: A Systematic Review and Meta-Analysis

**DOI:** 10.3390/ijms27093950

**Published:** 2026-04-29

**Authors:** Sheran P. W. Fernando, Prakash V. A. K. Ramdass

**Affiliations:** Department of Public Health and Preventive Medicine, St. George’s University School of Medicine, St. George FZ818, Grenada; sfernan3@sgu.edu

**Keywords:** adiponectin, adipokines, polycystic ovary syndrome, PCOS, adolescent girls

## Abstract

Polycystic ovary syndrome (PCOS) is a prevalent endocrine–metabolic disorder affecting 5.5–11.5% of women of reproductive age. While reduced adiponectin levels have been consistently demonstrated in adult women with PCOS, findings in adolescents remain less clearly defined. A systematic review and meta-analysis was conducted in accordance with PRISMA guidelines. PubMed, Embase, Scopus, and Google Scholar were searched from inception to 31 October 2025. Observational studies comparing adiponectin levels in post-pubertal adolescents with PCOS and controls were included. Data were pooled using random-effects meta-analysis in RStudio, with subgroup, sensitivity, heterogeneity, and publication bias analyses conducted. Eighteen studies comprising 1590 participants were analyzed. The pooled analysis demonstrated significantly lower adiponectin levels in adolescents with PCOS compared to controls (mean difference [MD]: −3.19 µg/mL; 95% CI: −4.90 to −1.49; *p* = 0.0010), with substantial heterogeneity (I^2^ > 90%). Subgroup analyses by diagnostic criteria, geographic region, study design, and weight status consistently showed reduced adiponectin levels in PCOS, with no significant subgroup differences. In conclusion, adolescents with PCOS exhibit significantly lower adiponectin levels, consistent with findings in adult populations. These results support an early association between adiponectin dysregulation and PCOS, although further longitudinal studies are needed to clarify clinical utility.

## 1. Introduction

Polycystic ovary syndrome (PCOS) is a common and complex endocrine–metabolic disorder affecting women of reproductive age, characterized by chronic anovulation, hyperandrogenism, and polycystic ovarian morphology [1]. It is estimated to affect approximately 5.5–11.5% of women worldwide, making it one of the most prevalent endocrine conditions in this population [2]. Beyond its reproductive manifestations, PCOS is strongly associated with insulin resistance, obesity, dyslipidemia, and an increased risk of type 2 diabetes mellitus and cardiovascular disease, highlighting its importance as a major public health concern [3,4,5].

Adiponectin, an adipocyte-derived hormone with insulin-sensitizing, anti-inflammatory, and anti-atherogenic properties, has emerged as a key regulator of metabolic homeostasis [6]. Lower circulating adiponectin levels have been consistently linked to obesity, insulin resistance, and cardiometabolic dysfunction—features that are highly prevalent in women with PCOS [7]. Consequently, adiponectin has been proposed as a potential mechanistic link between adipose tissue dysfunction and the metabolic abnormalities observed in PCOS [8].

Several large-scale meta-analyses in adult women have demonstrated a robust association between PCOS and reduced circulating adiponectin concentrations [9,10]. However, adolescents represent a biologically distinct population undergoing pubertal hormonal transitions, evolving insulin sensitivity, and dynamic changes in adipose tissue biology [11,12]. Adolescence represents a unique developmental window characterized by physiological pubertal insulin resistance, rising sex hormone concentrations, and rapid adipose tissue remodeling [13]. These normal maturational changes may either mask or amplify PCOS-related metabolic alterations, making it essential to examine adipokine profiles specifically within this age group rather than extrapolating from adult data.

Despite robust evidence of hypoadiponectinemia in adult women with PCOS [14], no meta-analysis has specifically focused on adolescent populations. It therefore remains unclear whether reduced adiponectin levels are already present during adolescence or emerge later as a consequence of prolonged disease. Establishing this is important, as early identification of hypoadiponectinemia could inform metabolic risk stratification and targeted preventive interventions. To address this gap, we conducted a systematic review and meta-analysis of observational studies comparing adiponectin levels in post-pubertal female adolescents with PCOS and controls.

## 2. Materials and Methods

### 2.1. Study Protocol and Registration

This systematic review and meta-analysis was conducted following the Preferred Reporting Items for Systematic Review and Meta-Analyses (PRISMA) guidelines and protocols (PRISMA-P) statement [15]. The study protocol was prospectively registered in the PROSPERO database (University of York, York, UK) [CRD420250654706]. Minor deviations from the original protocol were made, including the addition of subgroup analyses and sensitivity analyses. These modifications were implemented to better explore sources of heterogeneity and were not expected to influence the primary conclusions. The research question was based on the PECO (Population, Exposure, Control, Outcome) framework: “In post-pubertal female adolescents (population), are serum adiponectin levels (exposure) different in adolescents with PCOS (outcome) than in adolescents without PCOS (control)?”

### 2.2. Eligibility Criteria, Information Sources, Search Strategy

**Figure 1:**
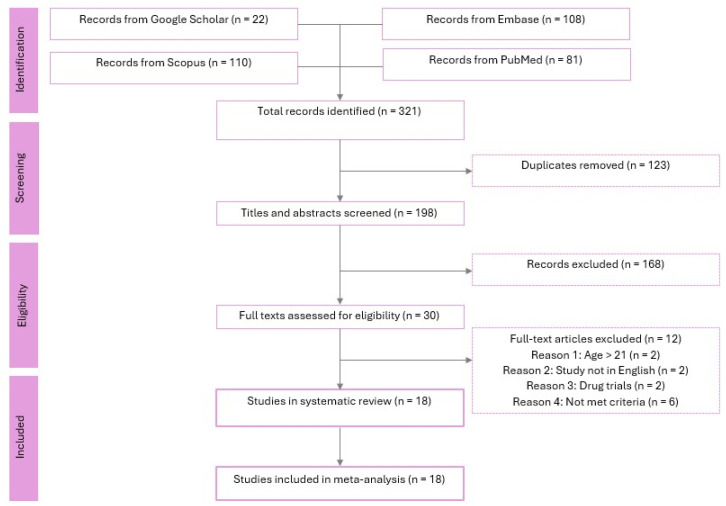
PRISMA flow chart.

A systematic search of the literature was performed from inception to 31 October 2025 using Embase, Scopus, PubMed, and Google Scholar databases. The search strategy employed the following keywords: (“adiponectin” OR “adipokines”) AND (“adolescent” OR “girls”) AND (“polycystic ovary syndrome” OR “PCOS”). Citations retrieved from each database were compiled and imported into Zotero reference management software, version 8.0.4. After duplicate removal, the titles and abstracts of remaining records were screened for eligibility. Full texts of potentially relevant studies were then assessed against predefined criteria. Two reviewers (S.F. and P.R.) carried out all screening. Details of the screening process are shown in the PRISMA flow chart in Figure 1.

Inclusion criteria comprised original case–control, cross-sectional, and cohort studies involving female adolescents diagnosed with PCOS. Diagnosis of PCOS was required to be based on the Rotterdam criteria [16], the National Institute of Health (NIH) criteria [17], or other clearly stated diagnostic criteria. Studies were excluded if they were case reports, reviews, animal studies, abstracts, drug trials, or conference presentations, as well as studies not published in English or Spanish. Articles published in Spanish were translated into English by a translator. No restrictions were applied regarding the year of publication.

### 2.3. Data Extraction and Assessment of Bias

Data were extracted and transferred into a Microsoft Excel file using a standardized template with the following column headings: study, country, study design, sample size, PCOS diagnostic criteria, and adiponectin levels (including mean and standard deviation [SD]), medication use, lifestyle intervention such as diet and exercise, inflammatory markers, and potential confounders. In addition, the weight according to BMI status of participants was noted, whether normal weight, obese, or mixed categories. For studies that reported adiponectin values as median and interquartile range, the mean and SD were estimated using the method described by Wan et al. [18]. Several included studies [19,20,21,22,23,24] reported serum adiponectin concentrations in ng/mL with mean values between 1.7 and 18 ng/mL. Based on reference data from Lausten-Thomsen et al. [25], normal total adiponectin levels in healthy, non-obese adolescents range from approximately 1.3 to 16.0 µg/mL (2.5th–97.5th percentiles for a 16-year-old); consequently, studies reporting values in the ng/mL range were considered to have a unit error, and their numerical values were treated as µg/mL without conversion. Studies reporting mg/L (Rossi et al. [26]) were treated as numerically equivalent to µg/mL. This approach ensures physiological plausibility and enables consistent cross-study comparison.

To ensure analytical precision, the extracted data were further stratified into subcategories based on the type of adiponectin measurement: total adiponectin or high molecular weight (HMW) adiponectin. To minimize the risk of duplicate data, studies were carefully examined for potential overlap in study populations. Particular attention was given to studies originating from the same research groups, institutions, or timeframes.

The methodological quality of all included studies was independently assessed by two reviewers (S.F. and P.R.) using the Newcastle–Ottawa Scale (NOS) [27]. The NOS is a validated tool for evaluating the quality of nonrandomized studies in meta-analyses and systematic reviews, with assessments based on three key domains: selection of study groups, comparability of groups, and ascertainment of exposure or outcome. Overall scores were recorded for each study.

### 2.4. Data Synthesis and Analysis

Data analysis was performed using RStudio software, version 4.4.1., to conduct pooled analyses and generate forest plots for assessing heterogeneity. The primary packages used included meta for meta-analysis, metafor for additional statistical modeling and meta-regression, readxl for data import, and dplyr for data management. A random-effects model was applied to account for the high heterogeneity among patient populations, attributed to differences in factors such as age, PCOS subtypes, and diagnostic criteria. This approach provides a more conservative estimate of the overall mean difference. Additionally, sub-group meta-analysis was performed according to PCOS criteria, study region, study design, and weight status.

Heterogeneity was assessed using Cochran’s Q test and the I^2^ statistic. A random-effects model with REML estimator and Hartung–Knapp adjustment was applied. An I^2^ value of 50% or higher was interpreted as indicating substantial variability, suggesting that the studies may not be estimating a common effect. To assess the robustness of the pooled estimates, multiple sensitivity analyses were conducted: leave-one-out analysis, influence diagnostics using the Baujat plot and formal influence statistics, including studentized residuals, Cook’s distance, DFFITS, covariance ratios, and leave-one-out heterogeneity estimates (τ^2^ and QE). Studies with standardized residuals exceeding ±2 were considered potential outliers and were excluded in a subsequent sensitivity analysis. Finally, sensitivity to effect size metric was assessed using standardized mean differences (SMD), and subgroup-specific analyses were performed by study design.

Publication bias was examined through a visual inspection of funnel plots, supplemented by Egger’s test [28] and Begg’s test [29]. Statistical significance was defined as a *p*-value of less than 0.05 for all analyses.

## 3. Results

### 3.1. Study Selection

Our search of PubMed, Embase, Scopus, and Google Scholar yielded a total of 321 records. After removing 123 duplicates, 198 titles and abstracts were screened for eligibility. Of these, 168 records were excluded based on title and abstract review, leaving 30 full-text articles to be assessed for inclusion. Following full-text review, 12 articles were excluded for the following reasons: age > 21 years (*n* = 2), study not in English or Spanish (*n* = 2), drug trials (*n* = 2), and failure to meet diagnostic or study criteria (*n* = 6). Ultimately, 18 studies [19,20,21,22,23,24,26,30,31,32,33,34,35,36,37,38,39,40] met all inclusion criteria and were included in both the systematic review and meta-analysis.

### 3.2. Study Characteristics

A total of 18 studies published between 2008 and 2025 were included in our analysis. The studies were conducted across nine countries, with the highest numbers from the USA (*n* = 7) and Türkiye (*n* = 5). Fifteen studies employed a case–control design, with the exception of two prospective cohort studies and one cross-sectional study. Sample sizes ranged from 30 to 363 participants, with a total of 1590 participants across all studies.

Regarding diagnostic criteria for PCOS, the Rotterdam criteria [16] were most frequently applied (*n* = 8), followed by the NIH criteria (*n* = 6) [17]. Two studies used the Endocrine Society Clinical Practice Guideline (ESCPG) [41], and two studies applied the International Evidence-based Guideline (IEG) [42]. One study [24] was published in Spanish, and was translated into English by a bilingual translator.

Overall, adjustment for confounders was largely consistent and centered on metabolic, anthropometric, and hormonal factors, particularly body mass index (BMI)/adiposity, insulin resistance (e.g., HOMA-IR or oral glucose tolerance test [OGTT]), lipid profiles, and androgen levels, with some studies additionally accounting for variables such as hepatic fat, blood pressure, diet, or socioeconomic status. In contrast, inflammatory markers were assessed less frequently and inconsistently; only a subset of studies reported biomarkers such as C-reactive protein (CRP), while a few evaluated cytokines including IL-6 and TNF-α, and others reported markers like IL-10, TGF-β, VCAM-1, or PAI-1. Notably, most studies did not incorporate inflammatory markers as covariates or outcomes.

Across the included studies, adiponectin was predominantly measured as fasting serum total adiponectin, indicating strong methodological consistency. Only one study by Cankaya et al. [33] assessed HMW adiponectin, while another study by Güven et al. [19] incorporated dynamic measurements during an oral glucose tolerance test. Additionally, the study by Whooten et al. [39] reported a derived adiponectin-to-leptin ratio rather than raw adiponectin levels. These variations were limited but may contribute to minor heterogeneity in the pooled analysis.

Reporting of therapeutic interventions was minimal across the included studies. Only one study evaluated a pharmacologic intervention that included metformin, while the remaining studies did not report treatment status. Lifestyle-related interventions such as diet or physical activity were not assessed as exposure variables, with only isolated instances of short-term standardization prior to metabolic testing rather than true intervention reporting.

Study quality, assessed using the Newcastle–Ottawa Scale (NOS) [27], yielded scores ranging from 4.5 to 8.5. The majority of studies (*n* = 11) scored 7 or above, indicating high methodological quality. Details of the study characteristics are shown in Table 1.

### 3.3. Meta-Analysis of MD in Adiponectin Based on PCOS Criteria

**Figure 2:**
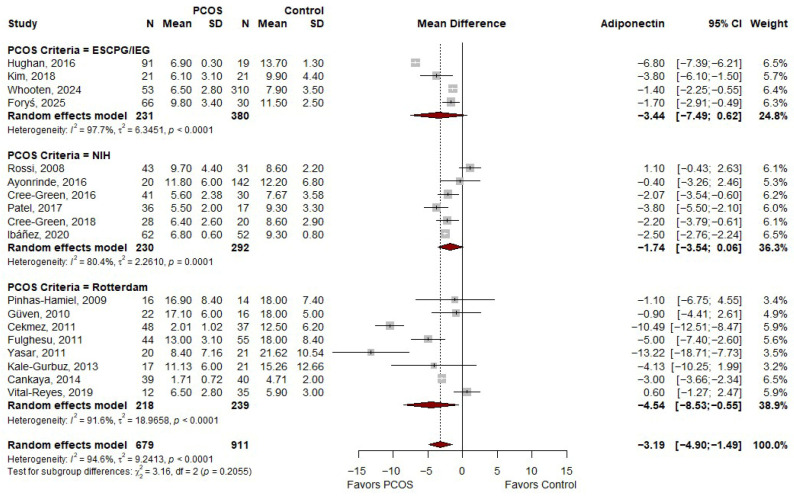
Forest plot of MD in adiponectin levels between PCOS and controls, according to PCOS criteria [19,20,21,22,23,24,26,30,31,32,33,34,35,36,37,38,39,40]. PCOS—Polycystic Ovary Syndrome; N—Sample Size; SD—Standard Deviation; CI—Confidence Interval.

The meta-analysis presented in Figure 2 shows that adiponectin levels were significantly lower in individuals with PCOS compared to controls, with an overall MD of −3.19 µg/mL (95% CI: −4.90 to −1.49) and *p* = 0.0010. When stratified by diagnostic criteria, studies using the ESCPG/IEG criteria demonstrated a non-significant reduction in adiponectin levels (MD: −3.44; 95% CI: −7.49 to 0.62) with considerable heterogeneity (I^2^ = 97.7%). Studies applying the NIH criteria also showed a reduction in adiponectin levels that approached but did not reach statistical significance (MD: −1.74; 95% CI: −3.54 to 0.06), with moderate-to-high heterogeneity (I^2^ = 80.4%). In contrast, studies using the Rotterdam criteria demonstrated a statistically significant decrease in adiponectin levels in PCOS (MD: −4.54; 95% CI: −8.53 to −0.55), although substantial heterogeneity was also observed (I^2^ = 91.6%). Despite these differences, there was no statistically significant subgroup effect based on diagnostic criteria (*p* for subgroup differences = 0.2055), indicating that the overall reduction in adiponectin levels is consistent across the different PCOS diagnostic frameworks.

### 3.4. Meta-Analysis of MD in Adiponectin Based on Study Region

**Figure 3:**
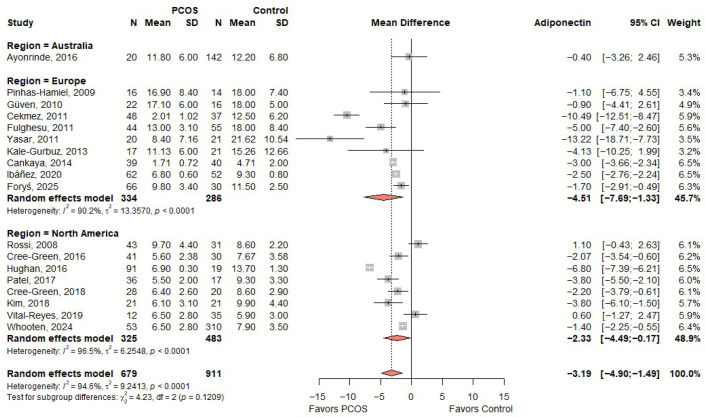
Forest plot of MD in adiponectin levels between PCOS and controls, according to study region [19,20,21,22,23,24,26,30,31,32,33,34,35,36,37,38,39,40]. PCOS—Polycystic Ovary Syndrome; N—Sample Size; SD—Standard Deviation; CI—Confidence Interval.

When stratified by geographic region, the meta-analysis in Figure 3 showed that European studies reported a significant reduction in adiponectin levels among individuals with PCOS (MD: −4.51; 95% CI: −7.69 to −1.33), although there was considerable heterogeneity across studies (I^2^ = 90.2%). Similarly, studies conducted in North America demonstrated a significant decrease in adiponectin levels (MD: −2.33; 95% CI: −4.49 to −0.17), with considerable heterogeneity (I^2^ = 96.5%). In contrast, the single study from Australia did not show a statistically significant difference between PCOS and control groups (MD: −0.40; 95% CI: −3.26 to 2.46). Despite regional variations in effect size, there was no statistically significant difference between subgroups based on geographic region (*p* for subgroup differences = 0.1209), indicating that the overall inverse association between adiponectin levels and PCOS is consistent across regions.

### 3.5. Meta-Analysis of MD in Adiponectin Based on Study Design

**Figure 4:**
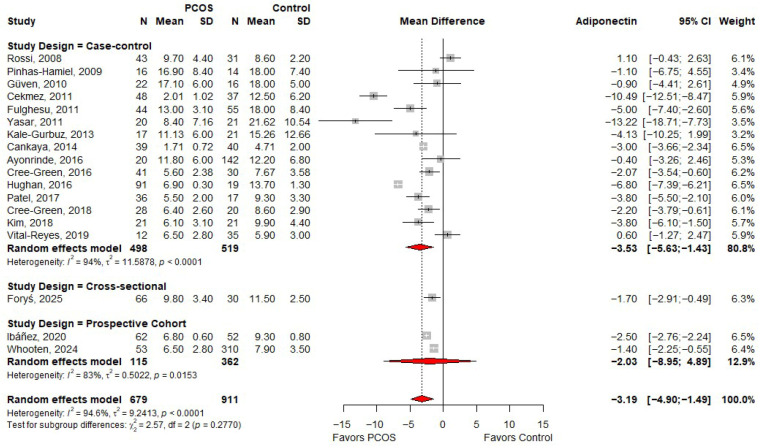
Forest plot of MD in adiponectin levels between PCOS and controls, according to study design [19,20,21,22,23,24,26,30,31,32,33,34,35,36,37,38,39,40]. PCOS—Polycystic Ovary Syndrome; N—Sample Size; SD—Standard Deviation; CI—Confidence Interval.

The meta-analysis in Figure 4 shows that, when stratified by study design, case–control studies demonstrated a significant reduction in adiponectin levels among individuals with PCOS (MD: −3.53; 95% CI: −5.63 to −1.43), although substantial heterogeneity was observed (I^2^ = 94%). The single cross-sectional study also reported lower adiponectin levels in PCOS (MD: −1.70; 95% CI: −2.91 to −0.49). In contrast, prospective cohort studies showed a non-significant reduction in adiponectin levels (MD: −2.03; 95% CI: −8.95 to 4.89), with moderate heterogeneity (I^2^ = 83%). Despite these variations, there was no statistically significant difference between study designs (*p* for subgroup differences = 0.2770), suggesting that the overall reduction in adiponectin levels among individuals with PCOS is consistent across different study types.

### 3.6. Meta-Analysis of MD in Adiponectin Based on Weight Status

**Figure 5:**
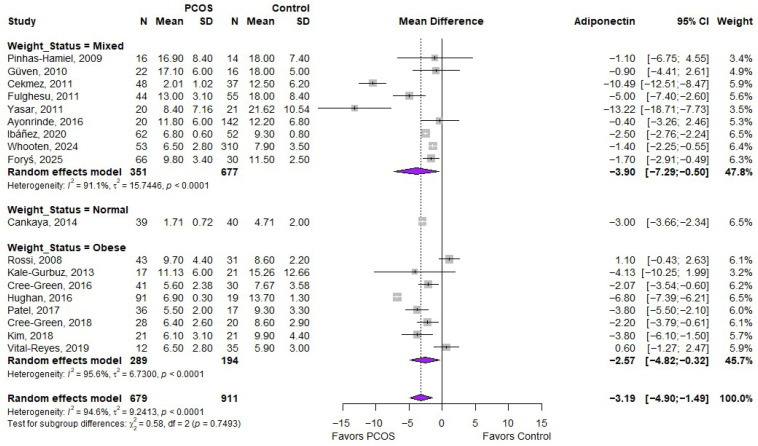
Forest plot of MD in adiponectin levels between PCOS and controls, according to weight status [19,20,21,22,23,24,26,30,31,32,33,34,35,36,37,38,39,40]. PCOS—Polycystic Ovary Syndrome; N—Sample Size; SD—Standard Deviation; CI—Confidence Interval.

The meta-analysis in Figure 5 shows that adiponectin levels were significantly lower in individuals with PCOS across all weight categories. Studies with mixed populations demonstrated a significant reduction (MD: −3.90; 95% CI: −7.29 to −0.50), as did the single normal-weight study (MD: −3.00; 95% CI: −3.66 to −2.34) and studies in obese populations (MD: −2.57; 95% CI: −4.82 to −0.32). Despite variations in effect size, there was no significant difference between subgroups (*p* = 0.7493), indicating that the inverse association between adiponectin and PCOS is consistent regardless of weight status, although heterogeneity across studies remained high.

### 3.7. Sensitivity Analysis

**Figure 6:**
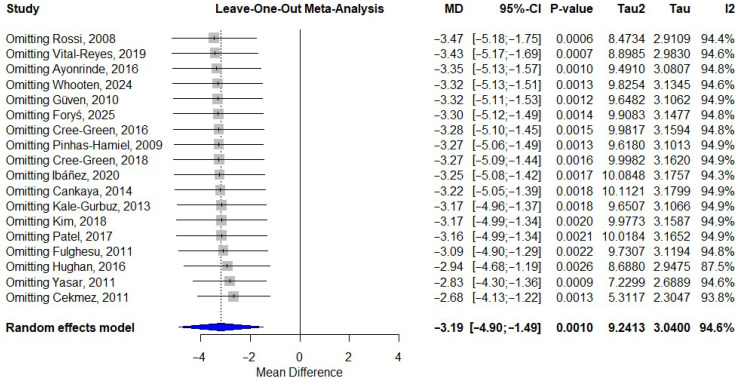
Leave-one-out sensitivity analysis [19,20,21,22,23,24,26,30,31,32,33,34,35,36,37,38,39,40]. MD—mean difference; CI—Confidence Interval.

**Figure 7:**
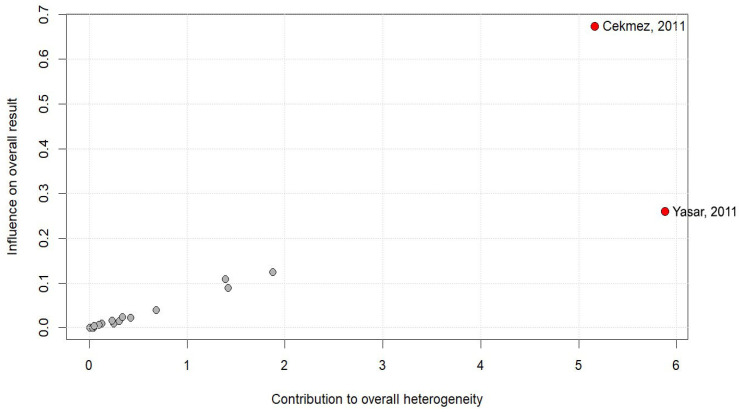
Baujat plot and influence diagnostics [19,20,21,22,23,24,26,30,31,32,33,34,35,36,37,38,39,40].

The leave-one-out analysis, shown in Figure 6, demonstrated that the pooled effect size remained stable regardless of the exclusion of any single study, with pooled mean differences ranging from −2.68 to −3.47. The Baujat plot and influence diagnostics, shown in Figure 7, identified Cekmez et al. [31] and Yasar et al. [21] as the most influential studies, contributing disproportionately to both heterogeneity and effect size. This was corroborated by high Cook’s distance values and large negative studentized residuals (−3.00 and −2.63, respectively). The exclusion of these outliers attenuated the pooled effect size from −3.19 (95% CI: −4.90 to −1.49) to −2.36 (95% CI: −3.49 to −1.23), while substantially reducing between-study heterogeneity (τ^2^ from 9.24 to 3.84), although I^2^ remained high (93.9%). When standardized mean differences were used, the pooled effect remained significant (SMD = −1.43, 95% CI: −2.70 to −0.16), supporting consistency across effect size metrics. Additionally, sensitivity analyses did not indicate disproportionate influence from any single study or cluster of studies, suggesting that potential overlap, if present, is unlikely to have materially altered the overall findings.

### 3.8. Publication Bias

**Figure 8:**
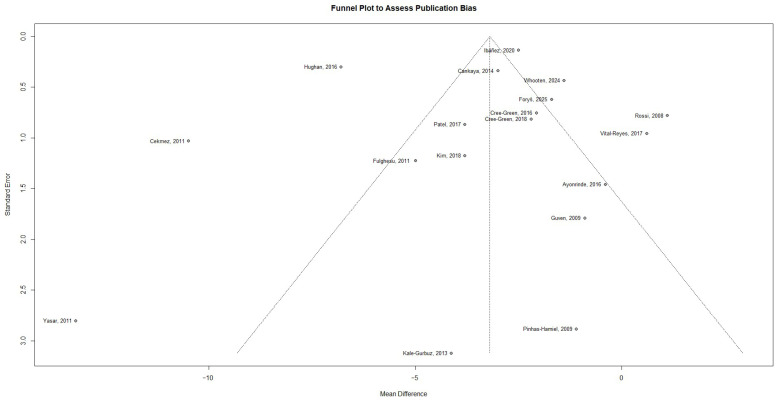
Funnel plot to assess publication bias [19,20,21,22,23,24,26,30,31,32,33,34,35,36,37,38,39,40].

Visual inspection of the funnel plot in Figure 8 suggests approximate symmetry, although some dispersion of studies is evident. Given the substantial heterogeneity observed across studies, this visual pattern should be interpreted with caution. Formal statistical tests for publication bias were non-significant, with Egger’s regression test [28] (t = −0.24, *p* = 0.81) and Begg’s rank correlation test [29] (z = −1.40, *p* = 0.16), showing no evidence of small-study effects. However, the presence of high heterogeneity and the limited number of studies reduce the power of these tests, and therefore the possibility of publication bias cannot be definitively excluded.

## 4. Discussion

In this systematic review and meta-analysis comprising 1590 adolescents, we found that post-pubertal girls with PCOS have significantly lower circulating adiponectin levels compared with their non-PCOS peers. The pooled estimate demonstrated a mean difference of −3.17 µg/mL, indicating a consistent and clinically meaningful reduction in adiponectin among adolescents with PCOS. Notably, 83% of the studies included reported lower adiponectin concentrations in the PCOS group. Collectively, these findings suggest that hypoadiponectinemia is already present in adolescence among girls with PCOS, supporting the hypothesis that adipose tissue dysfunction and early metabolic dysregulation are integral components of the syndrome from its earliest clinical manifestations [12,43].

The present findings are broadly consistent with previous meta-analyses conducted in adult populations, which have similarly reported significantly lower adiponectin levels in women with PCOS compared to controls [9,10,14]. These adult studies have consistently demonstrated an inverse association between adiponectin and PCOS, often attributing this relationship to underlying insulin resistance and adiposity [10]. Our findings extend this evidence to adolescent populations, suggesting that reduced adiponectin levels are evident early in the disease course, prior to the full manifestation of long-term metabolic complications.

However, compared with adult meta-analyses, the magnitude of effect in our study appears more variable, likely reflecting heterogeneity at the pubertal stage, duration of disease, and differences in metabolic profiles during adolescence. A major challenge in adolescent PCOS research is that diagnostic criteria developed for adults may not be directly applicable during puberty. In adult women, the Rotterdam criteria allow diagnosis based on two of three features: oligo-anovulation, hyperandrogenism, and polycystic ovarian morphology [16]. However, in adolescents, physiologic pubertal changes may mimic these features, including irregular menstrual cycles in the early post-menarcheal period, transient acne or hyperandrogenic signs, and multifollicular ovarian morphology [44]. For this reason, adolescent-focused guidelines recommend greater diagnostic caution, emphasizing persistent ovulatory dysfunction and hyperandrogenism while generally avoiding ultrasound-based ovarian morphology as a primary diagnostic criterion in early adolescence, given the substantial overlap with normal pubertal physiology [42]. In the present review, several included studies used adult-based criteria, particularly Rotterdam [19,20,21,31,32] or NIH [26,34,36,38] definitions, whereas more recent studies [35,37,39,40] used criteria more consistent with adolescent-specific recommendations. This variation may have introduced misclassification bias and contributed to heterogeneity; however, subgroup analysis by diagnostic criteria showed no significant subgroup differences, suggesting that diagnostic framework alone did not fully explain the observed association between lower adiponectin levels and PCOS.

Additionally, adult studies more frequently stratify by obesity and insulin resistance [45], whereas such stratification was limited in the current analysis due to inconsistent reporting. Together, these observations suggest that while hypoadiponectinemia is a consistent feature of PCOS across the lifespan, its determinants and clinical implications may differ between adolescents and adults, underscoring the need for age-specific investigations.

Adiponectin is closely linked to adiposity and metabolic status, particularly insulin resistance, both of which are highly prevalent in PCOS [46]. Therefore, the observed reduction in adiponectin levels may be partially influenced by underlying differences in obesity and metabolic profiles between cases and controls. However, the extent of this influence could not be fully assessed in the present analysis due to inconsistent reporting of BMI, adiposity measures, and metabolic parameters across studies. While some included studies accounted for BMI or evaluated insulin resistance [19,31], the lack of standardized reporting precluded formal adjustment or stratified meta-analysis. This represents a potential source of residual confounding and should be considered when interpreting the findings. Future studies should incorporate uniform reporting of anthropometric and metabolic variables to enable more refined analyses.

The included studies varied considerably in how adiposity was managed, which has important implications for interpreting the observed association between adiponectin and PCOS. Only one study [33] specifically compared normal-weight PCOS individuals with BMI-matched normal-weight controls, thereby minimizing the confounding effect of obesity. In contrast, a substantial proportion of studies [22,26,32,36,37] were conducted exclusively in overweight or obese populations, comparing obese PCOS participants with obese controls, reflecting metabolically enriched cohorts. A smaller number of studies [30,35] included both obese and lean groups but did not consistently stratify analyses by weight status, while many others reported mixed populations without clear BMI-based comparisons or subgroup analyses [20,31,34]. Collectively, this imbalance—with relatively few normal-weight comparisons and a predominance of overweight/obese cohorts—limits the ability to disentangle the independent effect of PCOS from that of adiposity on adiponectin levels and suggests that residual confounding by obesity remains a key consideration in interpreting these findings.

Adiponectin plays a central role in metabolic regulation by enhancing insulin sensitivity through the activation of key signaling pathways, including adenosine monophosphate-activated protein kinase (AMPK) and peroxisome proliferator-activated receptor alpha (PPAR-α) [47]. Activation of these pathways promotes glucose uptake in skeletal muscle and increases fatty acid oxidation in liver and peripheral tissues, thereby improving overall metabolic efficiency [48]. During normal puberty, adolescents experience a transient, physiologic reduction in insulin sensitivity, likely driven by hormonal changes associated with growth and sexual maturation [49]. In most individuals, this pubertal insulin resistance is temporary and resolves with completion of puberty. However, in girls with PCOS, reduced adiponectin levels may amplify this physiologic shift, tipping it toward a more sustained and pathologic state of insulin resistance due to underlying adipose tissue dysfunction [50]. Consequently, hypoadiponectinemia in adolescence may not merely reflect existing metabolic abnormalities but may also be associated with early alterations in cardiometabolic risk, potentially preceding the development of overt dysglycemia or type 2 diabetes.

A growing body of evidence supports a bidirectional relationship between hyperandrogenism and adipose tissue dysfunction in PCOS [51]. Elevated androgen levels may directly contribute to adverse changes in body fat distribution, promoting visceral adiposity and impairing normal adipocyte differentiation and function [10]. Visceral fat, in turn, is metabolically active and associated with reduced secretion of protective adipokines such as adiponectin [10]. Lower adiponectin levels can exacerbate insulin resistance, leading to compensatory hyperinsulinemia [52]. Hyperinsulinemia then acts synergistically with luteinizing hormone to stimulate ovarian theca cells, further increasing androgen production [53]. This creates a self-perpetuating pathogenic cycle: insulin resistance leads to hyperinsulinemia, which drives androgen excess; androgen excess worsens adipose tissue dysfunction; dysfunctional adipose tissue further reduces adiponectin levels, thereby reinforcing insulin resistance [53]. In adolescents with PCOS, this cycle may be established early, contributing to the persistence and progression of both reproductive and metabolic abnormalities.

Adolescence is characterized by the activation and maturation of the hypothalamic–pituitary–ovarian (HPO) axis, resulting in dynamic fluctuations in gonadotropins, estrogen, and androgens [54]. These hormonal shifts not only regulate reproductive function but also influence adipose tissue biology and adipokine secretion. Estrogen is generally associated with a more favorable metabolic profile, promoting subcutaneous fat deposition and supporting beneficial adipokine patterns, including relatively higher adiponectin levels [55]. In contrast, androgen excess—an established hallmark of PCOS—may adversely affect adipocyte function and suppress adiponectin secretion [51]. During puberty, when endocrine systems are still maturing, sustained exposure to elevated androgen levels may disrupt normal metabolic adaptation and adipose tissue development. Early hyperandrogenism during these critical developmental windows could therefore contribute to long-term metabolic programming, predisposing adolescents with PCOS to persistent insulin resistance and increased cardiometabolic risk in adulthood [56].

Adolescents with PCOS frequently demonstrate altered body composition characterized by increased central or visceral adiposity, even in the presence of a normal body mass index [57]. This suggests that BMI alone may underestimate underlying metabolic risk in this population. Visceral fat is metabolically active and more strongly associated with insulin resistance, inflammation, and adverse cardiometabolic outcomes than subcutaneous fat [58,59]. Importantly, visceral adipose tissue is linked to reduced adiponectin secretion, contributing to impaired insulin sensitivity [60]. Therefore, circulating adiponectin levels may reflect qualitative alterations in adipose tissue function and distribution rather than simply total fat mass. The early accumulation of visceral fat during adolescence may represent a key mechanistic pathway through which PCOS confers increased long-term risk of type 2 diabetes and cardiovascular disease, reinforcing the importance of early metabolic assessment beyond traditional anthropometric measures.

Adiponectin exerts important anti-inflammatory and vasculoprotective effects, including suppression of pro-inflammatory cytokine production and inhibition of endothelial dysfunction [61]. By enhancing nitric oxide bioavailability and reducing oxidative stress, adiponectin contributes to the maintenance of vascular integrity and metabolic homeostasis. Reduced adiponectin levels, as observed in adolescents with PCOS, may therefore facilitate the development of low-grade chronic inflammation—a well-recognized feature of the syndrome [62]. This inflammatory milieu, combined with insulin resistance and central adiposity, may initiate early endothelial and metabolic disturbances. Adolescence may represent a critical developmental window during which these early inflammatory and metabolic alterations become biologically embedded, potentially programming long-term susceptibility to type 2 diabetes mellitus and cardiovascular disease. Identifying hypoadiponectinemia during this period may thus provide insight into early cardiometabolic risk trajectories in girls with PCOS.

From a developmental origin and life-course perspective, our findings suggest that the metabolic disturbances associated with PCOS may emerge much earlier than traditionally recognized. The presence of hypoadiponectinemia during adolescence indicates that adipose tissue dysfunction and insulin resistance are not merely late complications of longstanding disease but may represent early pathophysiologic features of the syndrome [63]. This aligns with the concept that cardiometabolic risk trajectories are established during critical developmental windows, including puberty, when hormonal, metabolic, and adipose tissue changes are highly dynamic. Early alterations in adipokine regulation may therefore contribute to long-term metabolic programming, increasing susceptibility to type 2 diabetes and cardiovascular disease in adulthood. These findings underscore the importance of early metabolic screening in adolescents with PCOS, and support timely lifestyle and therapeutic interventions aimed at modifying risk before irreversible cardiometabolic sequelae develop.

The observed reduction in adiponectin levels among adolescents with PCOS has important clinical implications. Hypoadiponectinemia may be associated with early metabolic risk, and could help identify girls who are at increased risk for insulin resistance and future cardiometabolic complications, although this relationship remains observational and requires further longitudinal validation [64]. In addition, adiponectin represents a potential therapeutic target, as interventions that improve insulin sensitivity may also restore more favorable adipokine profiles [65,66]. Lifestyle strategies such as structured physical activity, dietary modification, and weight management have been shown to increase adiponectin levels and improve metabolic parameters. Similarly, insulin-sensitizing agents, including metformin, may enhance adiponectin concentrations while addressing underlying insulin resistance [67]. Emphasizing early prevention and targeted metabolic intervention during adolescence may therefore help modify long-term cardiometabolic trajectories and reduce the burden of type 2 diabetes and cardiovascular disease in women with PCOS.

Taken together, these findings suggest that adiponectin dysregulation is not merely a consequence of long-standing metabolic disease but may represent an early pathophysiologic feature of PCOS emerging during pubertal maturation.

Several important limitations should be considered. First, substantial heterogeneity was observed across studies, which was not fully explained by subgroup analyses, indicating the presence of unmeasured confounding factors. Second, the majority of included studies were observational and cross-sectional in design, limiting causal inference. Third, reporting of key variables such as BMI, adiposity measures, and metabolic parameters was inconsistent, precluding formal adjustment or stratified meta-analysis based on these factors. Fourth, there was an imbalance in study populations, with a predominance of overweight or obese cohorts and relatively few studies involving normal-weight participants, limiting the ability to isolate the independent effect of PCOS from that of adiposity. Finally, the modest number of included studies reduced the statistical power for subgroup analyses and publication bias assessment.

Despite these limitations, our findings reinforce and extend evidence previously demonstrated in adult women with PCOS, suggesting that reduced adiponectin is an early and persistent feature of the disorder. Future research should prioritize longitudinal cohort studies in adolescent populations to clarify the temporal relationship between adiponectin levels and the development of metabolic dysfunction in PCOS. Standardized reporting of anthropometric and metabolic variables, including BMI, body composition, and insulin resistance indices, is essential to enable more refined analyses. In addition, future studies should incorporate stratified analyses by weight status and metabolic phenotype to better disentangle the independent contributions of PCOS and adiposity. Harmonization of assay methods for adiponectin measurement would also improve comparability across studies. Finally, larger, multicenter studies with diverse populations are needed to reduce heterogeneity and enhance the generalizability of findings.

## 5. Conclusions

This systematic review and meta-analysis demonstrates that post-pubertal adolescent girls with PCOS have significantly lower circulating adiponectin levels compared with controls, suggesting that hypoadiponectinemia is an early feature of the syndrome. However, substantial unexplained heterogeneity, inconsistent adjustment for adiposity, and the absence of prospective data preclude the establishment of a diagnostic threshold or any clinical recommendation for adiponectin measurement in routine practice. At present, the evidence does not support the use of adiponectin as a clinical biomarker for screening, diagnosis, or risk stratification in adolescents with PCOS. Rather, adiponectin should remain an investigational tool for mechanistic and longitudinal studies. Future prospective research should determine whether baseline adiponectin levels predict incident metabolic dysfunction independent of BMI and whether early intervention can favorably modify adipokine profiles and long-term cardiometabolic outcomes.

## Figures and Tables

**Table 1:** Characteristics of included studies.

Study	Country	Study Design	Criteria for PCOS	Confounders	Inflammatory Markers	Sample Size	NOS Score
Rossi, 2008 [26]	USA	Case–Control	NIH	BMI, MetS, glucose, lipids	PAI-1	74	8
Pinhas-Hamiel, 2009 [30]	Israel	Case–Control	Rotterdam	BMI, insulin, androgens	-	30	4.5
Güven, 2010 [19]	Türkiye	Case–Control	Rotterdam	BMI, insulin resistance, OGTT, lipids	-	38	8
Cekmez, 2011 [31]	Türkiye	Case–Control	Rotterdam	Insulin resistance, adipocytokines	-	85	5
Fulghesu, 2011 [20]	Italy	Case–Control	Rotterdam	Insulin resistance (HOMA), BMI matching	IL-6, TNF-α, IL-10, TGF-β	99	8.5
Yasar, 2011 [21]	Türkiye	Case–Control	Rotterdam	BMI, hormonal profile (androgens)	-	41	7
Kale-Gurbuz, 2013 [32]	Türkiye	Case–Control	Rotterdam	BMI, hormonal parameters	-	38	4.5
Cankaya, 2014 [33]	Türkiye	Case–Control	Rotterdam	BMI, OGTT, insulin resistance	-	79	7
Ayonrinde, 2016 [34]	Australia	Case–Control	NIH	Adiposity, insulin resistance, testosterone	CRP	162	7
Cree-Green, 2016 [22]	USA	Case–Control	NIH	BMI, hepatic fat, insulin sensitivity	-	71	6
Hughan, 2016 [35]	USA	Case–Control	ESCPG	BMI, lipids, blood pressure, insulin sensitivity	CRP, VCAM-1	110	8
Patel, 2017 [36]	USA	Case–Control	NIH	BMI, controlled diet, insulin resistance	Cardiovascular markers	53	5
Cree-Green, 2018 [23]	USA	Case–Control	NIH	BMI, insulin resistance predictors	-	48	6.5
Kim, 2018 [37]	USA	Case–Control	ESCPG	BMI, fat oxidation, metabolic flexibility	-	42	4.5
Vital-Reyes, 2019 [24]	Mexico	Case–Control	Rotterdam	BMI, metabolic profile	IL-6, TNF-α	47	7
Ibáñez, 2020 [38]	Spain	Prospective Cohort	NIH *		CRP mentioned	114	7
Whooten, 2024 [39]	USA	Prospective Cohort	IEG	BMI, maternal PCOS, SES	Cardiometabolic markers	363	5.5
Foryś, 2025 [40]	Poland	Cross-sectional	IEG	BMI, adipokine profile, metabolic markers	-	96	8.5

PCOS—Polycystic Ovary Syndrome; NOS—Newcastle–Ottawa Scale; NIH—National Institute of Health; ESCPG—Endocrine Society Clinical Practice Guideline; IEG—International Evidence-based Guideline. * This study used adolescent-specific diagnostic criteria consistent with NIH. - No data was provided.

## Data Availability

Data can be obtained upon request. All data used can be found in the following databases: PubMed, Embase, Scopus, and Google Scholar.

## Abbreviations

The following abbreviations are used in this manuscript:

PCOS	Polycystic Ovary Syndrome
NOS	Newcastle–Ottawa Scale
MD	Mean Difference
CI	Confidence Interval
N	Sample Size
PRISMA	Preferred Reporting Items for Systematic Review and Meta-Analyses
NIH	National Institute of Health
ESCPG	Endocrine Society Clinical Practice Guideline
IEG	International Evidence-based Guideline
